# Single-cell analysis of B cell dysregulation in pediatric sepsis stratified by disease severity

**DOI:** 10.1038/s41598-025-34126-9

**Published:** 2025-12-31

**Authors:** Fahd Alhamdan, Stefano Gianoli, Koichi Yuki, Sophia Koutsogiannaki

**Affiliations:** 1 Department of Anesthesiology, Critical Care and Pain Medicine, Cardiac Anesthesia Division, Boston Childrens Hospital, 300 Longwood Ave, Boston, MA 02115 USA; 2 Department of Anaesthesia, Harvard Medical School, Boston, MA 02115 USA; 3 Department of Immunology, Harvard Medical School, Boston, MA 02115 USA; 4 Broad Institute of Harvard and MIT, 415 Main St, 02142 Cambridge, MA USA

**Keywords:** Diseases, Immunology

## Abstract

Sepsis is a life-threatening condition arising from a dysregulated host response to infection, characterized by a complex interplay between pro-inflammatory and anti-inflammatory immune mechanisms. B cells, that are key components of humoral immunity, are essential for antibody production, antigen presentation, and immune modulation. Sepsis is commonly associated with B cell lymphopenia, particularly in severe cases, as evidenced by a marked reduction in circulating B cells in both adult and pediatric patients. However, the mechanisms driving this depletion have not been completely understood, particularly in pediatric sepsis. In this study, we employed single-cell RNA sequencing (scRNA-seq) to profile peripheral blood leukocytes from pediatric patients with sepsis (three with mild and three with severe condition) alongside four healthy controls. Our analysis revealed a broad depletion of immune cells in sepsis, with B cells showing the most pronounced reduction and the strongest capacity to discriminate between mild and severe disease states. Subset-level analysis identified significant loss and transcriptional reprogramming of natural killer-like B cells (ΝΚΒ), MHC II^high^naïve, and MHC II^low^naïve B cells in severe sepsis, accompanied by signatures indicative of intrinsic and extrinsic apoptosis, disrupted differentiation, and enhanced migratory potential. Importantly, we identified and validated the population of NKB cells in both pediatric and adult cohorts, broadening the known landscape of B cell phenotypes in human sepsis. We also explored intercellular communications, focusing on B cell-neutrophil interactions, given the notable neutrophilia observed, revealing potential crosstalk that may contribute to the overall immune dysregulation in sepsis. Collectively, these findings offer new insights into the cellular and molecular drivers of immune dysfunction in pediatric sepsis, shedding light on B cell depletion as a central feature of disease severity. This work lays the foundation for future diagnostic and therapeutic strategies targeting B cell-mediated immunity in septic children. The study was registered on ClinicalTrials.gov (NCT04103268), with registration date on 2019-09-23, before the first patient was enrolled.

**Supplementary Information:**

The online version contains supplementary material available at 10.1038/s41598-025-34126-9.

## Background

 Sepsis is a life-threatening clinical syndrome driven by a dysregulated immune response to infection, leading to systemic inflammation, tissue injury, and ultimately multi-organ failure^1–3^. Despite decades of intense research, no targeted immunomodulatory therapy has proven effective, and clinical management remains largely supportive. The burden is particularly profound in pediatric populations, where severe sepsis is a leading cause of morbidity and mortality, accounting for over one-third of deaths in tertiary pediatric intensive care units (PICUs)^4–6^. Although improvements in early recognition and supportive care have reduced initial mortality^7^, many patients continue to succumb to late-stage complications, driven by persistent inflammation, profound immunosuppression, and recurrent infections^8,9^.

Immune dysregulation is a central hallmark of sepsis pathophysiology^10^. Immunotherapies have shown promise in other diseases, yet translating such approaches to sepsis has been limited by the inherent complexity of the immune response^11^. Sepsis often involves both an overwhelming pro-inflammatory response and a simultaneous or subsequent state of immune suppression. These opposing forces can coexist and fluctuate rapidly within the same patient, underscoring that sepsis is not a uniform clinical entity, but a syndrome of immune mis-regulation^12^. The immune system itself is not monolithic, and consists of a dynamic and diverse network of cell types with distinct phenotypes, functions, and activation states^13^. Traditional analytical methods often fail to resolve this complexity, concealing critical shifts in immune behavior that may drive disease progression^14^.

High-throughput transcriptomic analysis at the single-cell level provides a powerful framework to capture the cellular heterogeneity and molecular dynamics underpinning sepsis. By enabling detailed resolution of immune cell states, plasticity, and intercellular communication, single-cell approaches can uncover both pathogenic and protective immune programs, offering new opportunities for early diagnosis and therapeutic intervention^15^. We have previously applied single-cell transcriptomics to pediatric sepsis, generating the first high-resolution immune cell atlas across the acute and recovery phases^16^. This work revealed distinct alterations in immune cell frequencies and states, including shifts in neutrophil subpopulations, and a previously unappreciated enrichment of innate-like CD4⁺ T cells during the acute phase. This T-cell subpopulation was enriched in pediatric sepsis, along with fractions of plasma and memory B cells, compared to adult sepsis. These findings established a foundational map of immune perturbation in pediatric sepsis that warrants further investigation. To this end, in the present study, we seek to further define how immune responses differ between mild and severe sepsis. While broad immune dysregulation has been well described, the immune correlates of disease severity remain poorly understood^1^. A deeper understanding of how specific immune cell subsets, activation states, and intercellular networks correlate with disease progression and clinical severity could yield early prognostic markers and risk factors and uncover novel targets for therapeutic modulation^17^. Specific immune cell subsets, such as T cells, B cells, or neutrophils, may be selectively depleted, expanded, or reprogrammed in severe disease, while preserved or compensatory populations may characterize milder presentations. By capturing these differences at single-cell resolution, we aim to identify early molecular signatures that predict clinical deterioration, well before overt organ failure occurs.

Ultimately, this work seeks to bridge the gap between mechanistic immunology and clinical translation. By decoding the immune landscape across sepsis severity, we lay the groundwork for precision medicine approaches, enabling earlier intervention, risk-stratification and patient-specific therapy, and improved outcomes in a condition that continues to pose a major global health challenge. This personalized strategy is particularly important in sepsis, where timing and context of intervention are crucial.

## Methods

### Human sepsis and control subject enrollment

This study investigated leukocyte signatures in pediatric patients with sepsis and age-matched healthy controls, enrolled between May 2021 and January 2023. Ethical approval was obtained from the Institutional Review Board at Boston Children’s Hospital, and written informed consent was secured from parents or legal guardians of all participants. When appropriate, assent was also obtained from the patients themselves. The study was conducted in accordance with the Declaration of Helsinki and was registered on ClinicalTrials.gov (Trial ID: NCT04103268). Eligible participants were between 1 month and 18 years of age.

### Sample collection and leukocyte purification

Control subjects included otherwise healthy pediatric patients undergoing elective surgical procedures. Sepsis patients were enrolled based on a documented or suspected infection and a pediatric Sequential Organ Failure Assessment (pSOFA) sub-score of ≥ 2 in at least one organ system at the time of Intensive Care Unit (ICU) admission^18^. Blood was collected at the time of ICU admission.

Patients with congenital heart disease, malignancies, autoimmune disorders, organ transplantation, human immunodeficiency virus (HIV) infection, or those receiving corticosteroid therapy were excluded. At each timepoint, 1 mL of peripheral blood was drawn into a heparin-coated tube. Leukocytes were isolated at room temperature using Polymorphprep (ProteoGenix, Schiltigheim, France), which separates polymorphonuclear and mononuclear cells. Isolated leukocytes were immediately fixed using 10x Genomics Fixation Reagent (10×Genomics, Pleasanton, CA) and stored in liquid nitrogen until further processing for single-cell RNA sequencing.

### 3′ RNA library preparation and sequencing

Following rapid thawing in a 37 °C water bath, single-cell RNA libraries were prepared using the Chromium Single Cell 3′ Reagent Kit (10x Genomics), according to the manufacturer’s protocol. Cell suspensions were counted and loaded into the Chromium Controller to target the recovery of approximately 3,000 Gel Beads-in-Emulsion (GEMs) per sample. The resulting libraries were assessed for quality using the Agilent TapeStation (Agilent Technologies, Santa Clara, CA) and quantified with the Qubit 2.0 Fluorometer (Thermo Fisher Scientific, Waltham, MA). Pooled libraries were further quantified by qPCR (Applied Biosystems, Waltham, MA) before sequencing. Libraries were sequenced on an Illumina platform using a configuration compatible with the 10x Genomics recommended specifications.

### Single-cell RNA-Seq data analysis

Raw sequencing data were pre-processed using the Cell Ranger Software Suite (version 3.1.0; 10x Genomics). Base call (BCL) files were converted to FASTQ format and demultiplexed using the mkfastq command. Unique molecular identifiers (UMIs) and cell barcodes were deconvoluted, and reads were aligned to the GRCh38 human reference genome to generate digital gene expression (DGE) matrices.

Downstream analysis was performed using the Seurat R package (version 5.2.1). Cells with fewer than 200 detected genes and genes expressed in fewer than 3 cells were excluded. Cells with > 10% mitochondrial transcript content were filtered out to remove low-quality or apoptotic cells. Following metadata curation, individual Seurat objects were merged and normalized. Dimensionality reduction, clustering, and visualization followed the standard Seurat workflow. A clustering resolution of 0.1 was used to identify major leukocyte populations.

To account for batch effects and technical variability, data integration was performed using the Harmony package (version 1.2.3), correcting for donor identity, sequencing batch, gender, and age. Differentially expressed genes (DEGs) and cluster-specific markers were identified using Seurat’s FindMarkers and FindAllMarkers functions, employing the Wilcoxon rank-sum test. Cell-type annotations were based on canonical marker genes, and independently validated using resources such as Azimuth^19^, CellMarker 2.0^20^, and other annotation algorithms. Receiver operating characteristic (ROC) curve analyses were performed using the R package pROC (version 1.18.5) to evaluate the discriminative power of gene expression profiles between severe and mild sepsis conditions. For each cell type, the top 500 most variable genes were selected, and ROC curves were generated to assess their ability to distinguish between the two clinical groups.

Functional enrichment analyses, including biological processes and pathways, were performed using clusterProfiler (version 4.12.6) in conjunction with Gene Ontology Biological Process^21^. Functional phenotypes and activity scores were calculated using Seurat’s AddModuleScore function, with gene sets sourced from the literature and Gene Ontology database.

To visualize differential gene expression across cell states and phenotypes, the dittoPlotVarsAcrossGroups function from the dittoSeq package was used (version 1.16.0).

Cell-cell communication networks were analyzed using the CellChat R package (version 1.6.1) with default parameters. The normalized gene expression matrix and cell cluster annotations were used as inputs. CellChat infers intercellular signaling interactions based on a curated database of ligand-receptor pairs, allowing identification of potential autocrine and paracrine communication among clusters. Gene–pathway network analysis was performed using the R package *ggraph* (version 2.2.1), based on Gene Ontology Biological Process (GO-BP) terms to visualize the relationships between genes and their associated pathways.

### Flow cytometry analysis

Heparinized whole blood samples from pediatric and adult subjects were kept on ice immediately after collection. To minimize non-specific antibody binding, cells were incubated with Fc receptor blocking reagent (dilution 1:50; Miltenyi Biotec, Bergisch Gladbach, Germany) at 4 °C for 15 min. Surface staining was then performed using a 1:50 dilution of each of the following fluorochrome-conjugated monoclonal antibodies: PE-conjugated anti-human CD20, APC-conjugated anti-human CD159a, and FITC-conjugated anti-human CD3 (all from BD Biosciences, Franklin Lakes, NJ). Following staining, red blood cells were lysed using BD’s FACS Lysing Buffer according to the manufacturer’s instructions. The remaining leukocytes were washed with PBS, resuspended, and analyzed using a BD Accuri™ flow cytometer. Data were exported as FCS files and analyzed with FlowJo v10.9.1.

### Statistical analysis

Statistical analysis was performed using GraphPad Prism 9 software package (GraphPad Software Inc., San Diego, CA). The type of statistical methods used was described under each figure legend. *P* < 0.05 was considered statistically significant.

## Results

### Immune cell profiling in severe and mild pediatric sepsis

**Fig. 1:**
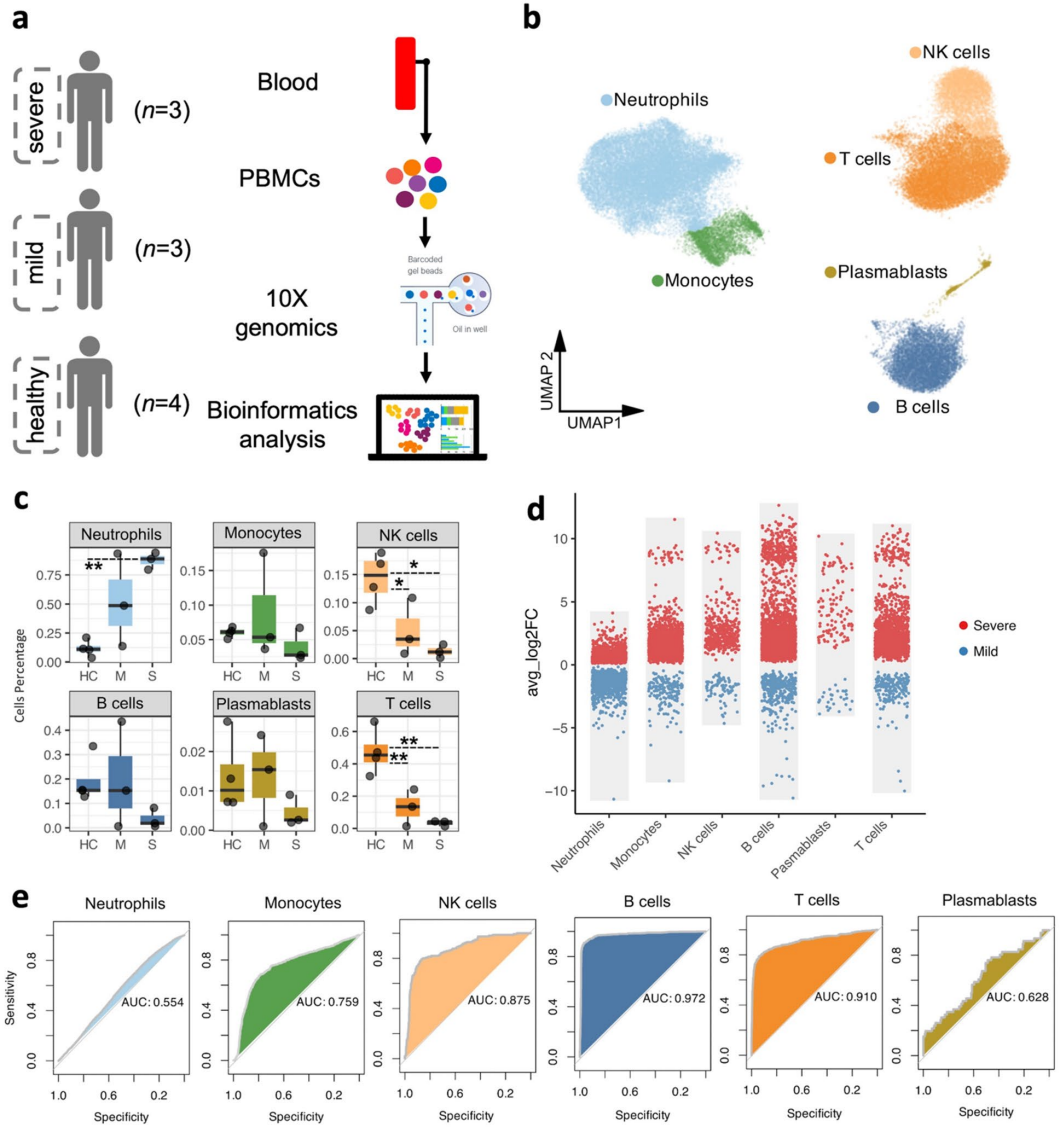
Major circulating leukocyte populations in pediatric sepsis. **a**. Schematic overview of the study design, including participants with severe sepsis (*n* = 3), mild sepsis (*n* = 3), and healthy controls (HC, *n* = 4). **b**. Uniform Manifold Approximation and Projection (UMAP) visualization of major circulating leukocyte subsets with annotated cell type identities. **c**. Boxplots illustrating the relative abundance of each leukocyte population across the three study groups. **d**. Scatter plots showing differentially expressed genes (DEGs) between severe and mild sepsis (highlighted in red and blue, respectively) within each major cell type. **e**. Receiver Operating Characteristic (ROC) curve analysis displaying the area under the curve (AUC) scores for each leukocyte population, assessing their predictive performance in distinguishing severe from mild sepsis.

Peripheral blood samples from three children with severe sepsis, three with mild sepsis at the time of ICU admission, and four healthy controls were used for this study (Fig. 1a). Clinical demographics and pSOFA scores are presented in Table 1. To capture transcriptomic alterations at single-cell resolution, we isolated peripheral blood leukocytes and performed single-cell RNA sequencing using the 10x Genomics Chromium platform. Across 10 datasets, we profiled a total of 56,178 cells, which were categorized into six major immune cell types (Fig. 1b; **Supp.** Figure 1a). Severe sepsis samples contained 3,560, 1,779, and 8,805 cells, respectively; mild sepsis samples contained 4,429, 4,952, and 4,155 cells; and healthy donor samples contained 2,789, 14,347, 2,633, and 4,788 cells, respectively (**Supp**. Table 1).

**Table 1:** Demographic data of sepsis and control subjects.

Subject	Age (year)	Gender	Diagnosis	pSOFA
Severe 1	1	Female	Bronchiolitis	6
Severe 2	15	Female	Toxic shock syndrome	12
Severe 3	3	Female	Aspiration pneumonia	7
Mild 1	0.7	Male	RSV infection	3
Mild 2	15	Male	Otomastoiditis	3
Mild 3	15	Male	Candidiasis	8
Healthy 1	0.3	Male	0	0
Healthy 2	10	Female	0	0
Healthy 3	6	Male	0	0
Healthy 4	0.8	Female	0	0

RSV, Respiratory Syncytial virus.

We next assessed differences in cell-type composition across the three groups (severe, mild, and healthy). Neutrophils were the only immune cells markedly elevated in both severe and mild sepsis compared to healthy controls, and the only cell population enriched in severe sepsis, compared to mild sepsis (Fig. 1c). In contrast, the proportions of T cells (both CD4⁺ T cells and CD8⁺ T cells), and NK cells were significantly reduced in both mild and severe sepsis, while monocytes, B-cells and plasmablasts were significantly reduced only in severe sepsis.

Differential gene expression analysis across all annotated cell types revealed that B cells showed the most pronounced transcriptional changes between severe and mild sepsis (Fig. 1d). This was further supported by ROC-AUC analysis, which demonstrated high discriminatory power in B cells from transcriptomic standpoint (AUC = 0.972) (Fig. 1e), while neutrophils showed minimal discrimination (AUC = 0.554) between the two sepsis groups. Collectively, these results, along with ROC-AUC scores for other immune cell types, suggest that adaptive immune cells, particularly B cells, play a central role in determining sepsis severity in pediatric patients.

### B cell subtypes in severe sepsis

**Fig. 2:**
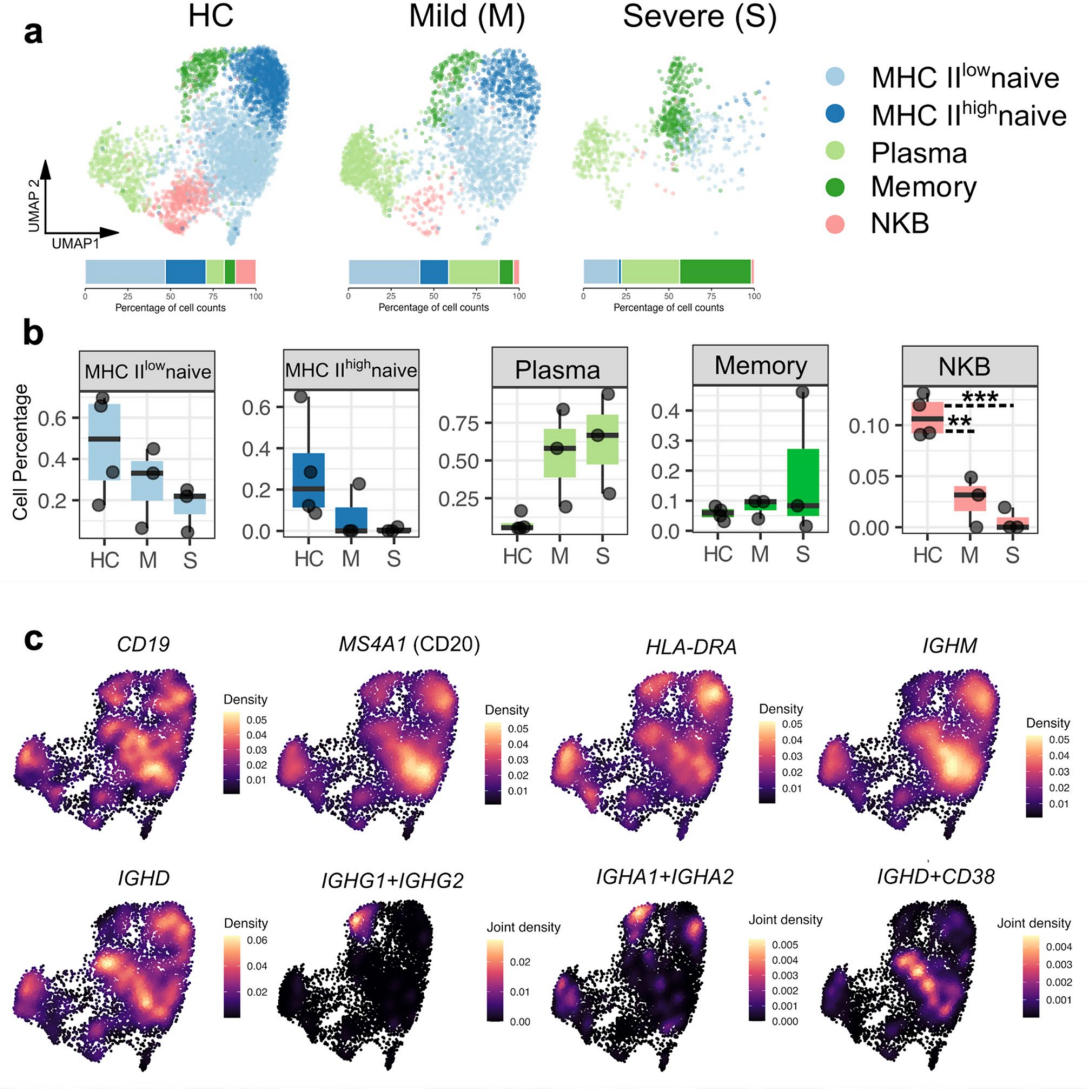
B cell subpopulations and their developmental trajectories in pediatric sepsis. **a**, UMAP visualization of B cells from all 10 study participants across the three study conditions, annotated by subpopulation. **b**, Boxplots showing the relative abundance of each B cell subpopulation across the three study groups. **c**, UMAP Density illustrating the expression of canonical B cell marker genes.

As previously noted, B cells exhibited the highest discriminatory power in distinguishing individuals with severe sepsis from those with mild disease. To gain a more detailed understanding of their gene expression profiles and underlying heterogeneity, we performed sub-clustering of B cells. This analysis revealed five distinct B cell subpopulations (Fig. 2a), each characterized by three marker genes (**Supp.** Figure 1b) based on the literature^22^. Among these subtypes, plasma cells showed a notable increase in proportion in severe sepsis, in contrast to the other B cell populations (Fig. 2b). To better characterize these subtypes, we visualized the expression of classical B cell markers such as CD19 and MS4A1 (CD20), the antigen presentation gene HLA-DRA, naïve B cell markers including IGHM and IGHD, memory B cell markers such as IGHG1, IGHG2, IGHA1, and IGHA2, and activated B cell markers including IGHD and CD38 (Fig. 2c).

### **Intrinsic apoptosis may underlie the depletion of MHC II**^**high**^**naïve B cells in pediatric severe sepsis**

**Fig. 3:**
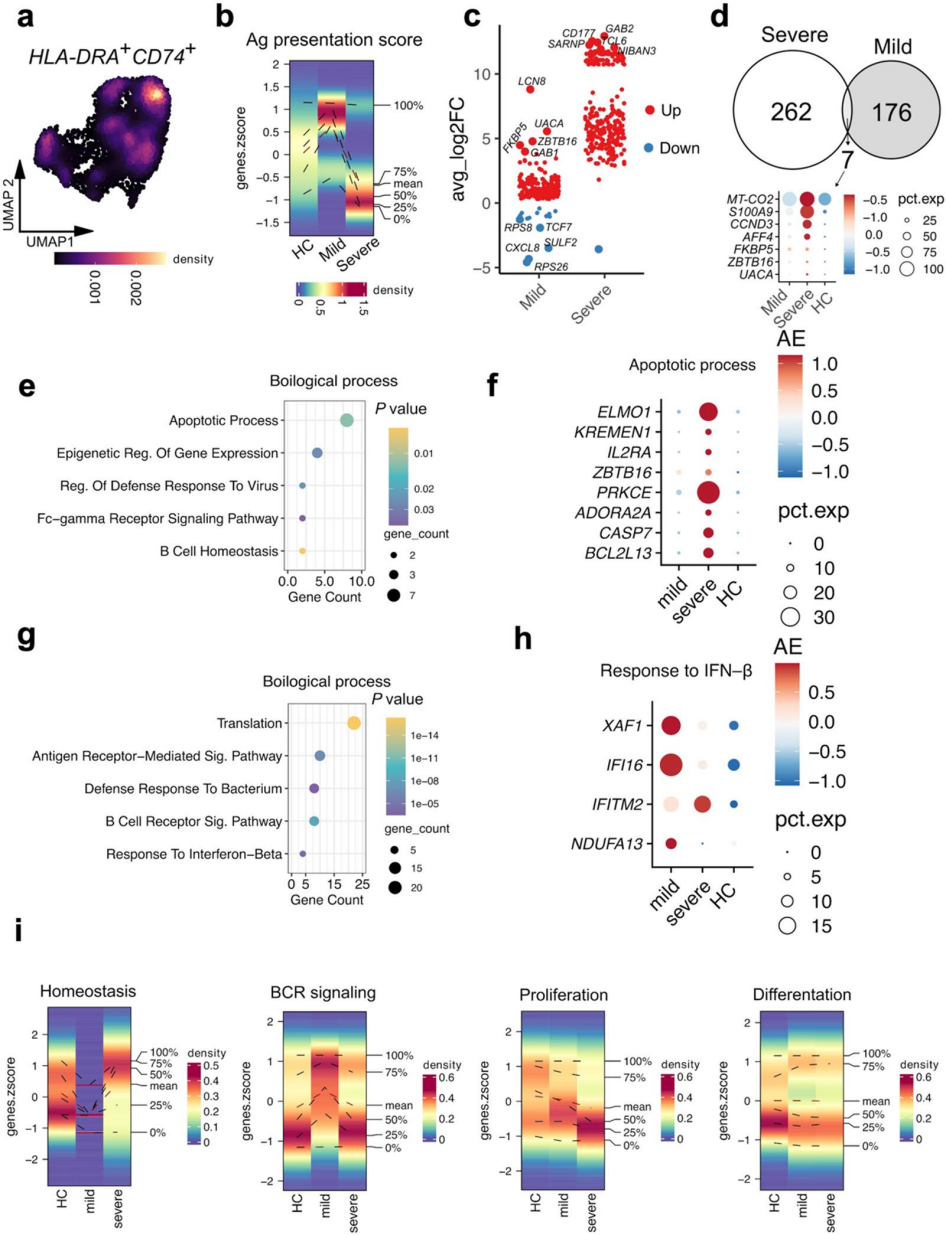
The depletion of MHC II^high^ naïve B cells in pediatric severe sepsis may be driven by intrinsic apoptosis. **a**. UMAP density plot showing the expression of antigen presentation–related genes in MHC II^high^ naïve B cells. **b**. Density heatmap illustrating antigen presentation scores in MHC II^high^ naïve B cells across the study groups. **c**. Scatter plots displaying differentially expressed genes (DEGs) in severe and mild sepsis compared to healthy controls (highlighted in red and blue, respectively). **d**. Venn diagram depicting the overlapping and unique DEGs identified in (**c**). **e**,** g**. Gene Ontology (GO) enrichment analysis of biological processes associated with DEGs in (**e**) severe sepsis and (**f**) mild sepsis. **f**,** h.** Dot plots highlighting the expression of representative genes from three selected biological processes identified in (**e**,** g**). **i.** Density heatmaps showing gene scores for canonical B cell processes across the three study conditions.

We next examined MHC II^high^naïve B cells, which were nearly depleted in severe sepsis. This prompted us to investigate their functional profile and transcriptional dynamics. MHC II^high^naïve B cells exhibited the highest expression of antigen presentation genes (Fig. 3a). However, the antigen presentation score was markedly reduced in severe sepsis compared to both mild sepsis and healthy controls (Fig. 3b).

To further explore these differences, we performed differential gene expression analysis comparing severe and mild sepsis to healthy donors (Fig. 3c). MHC II^high^naïve B cells from patients with severe sepsis displayed a distinct transcriptional profile, with a greater number of upregulated genes relative to mild sepsis, aligning with disease severity. Only seven differentially expressed genes overlapped between the severe and mild sepsis groups, all of which were more highly expressed in severe sepsis (Fig. 3d).

Gene Ontology enrichment analysis of biological processes revealed that intrinsic apoptotic pathway was the most significantly overrepresented in MHC II^high^naïve B cells from patients with severe sepsis (Fig. 3e), represented by *CASP7* and *BCL213* (Fig. 3f), potentially explaining their marked numerical reduction in this group. In contrast, MHC II^high^naïve B cells in mild sepsis exhibited enrichment for processes associated with activation, proliferation, and immune defense (Fig. 3g–h).

Finally, density analysis of functional scores in severe sepsis highlighted elevated homeostasis, which may reflect a compensatory response following significant cell loss. This was accompanied by a substantial decrease in proliferation and B cell receptor (BCR) signaling activity (Fig. 3i), suggesting a functional impairment of MHC II^high^naïve B cells in severe disease.

### Differentiation May define the fate of NKB cells in pediatric severe sepsis

**Fig. 4:**
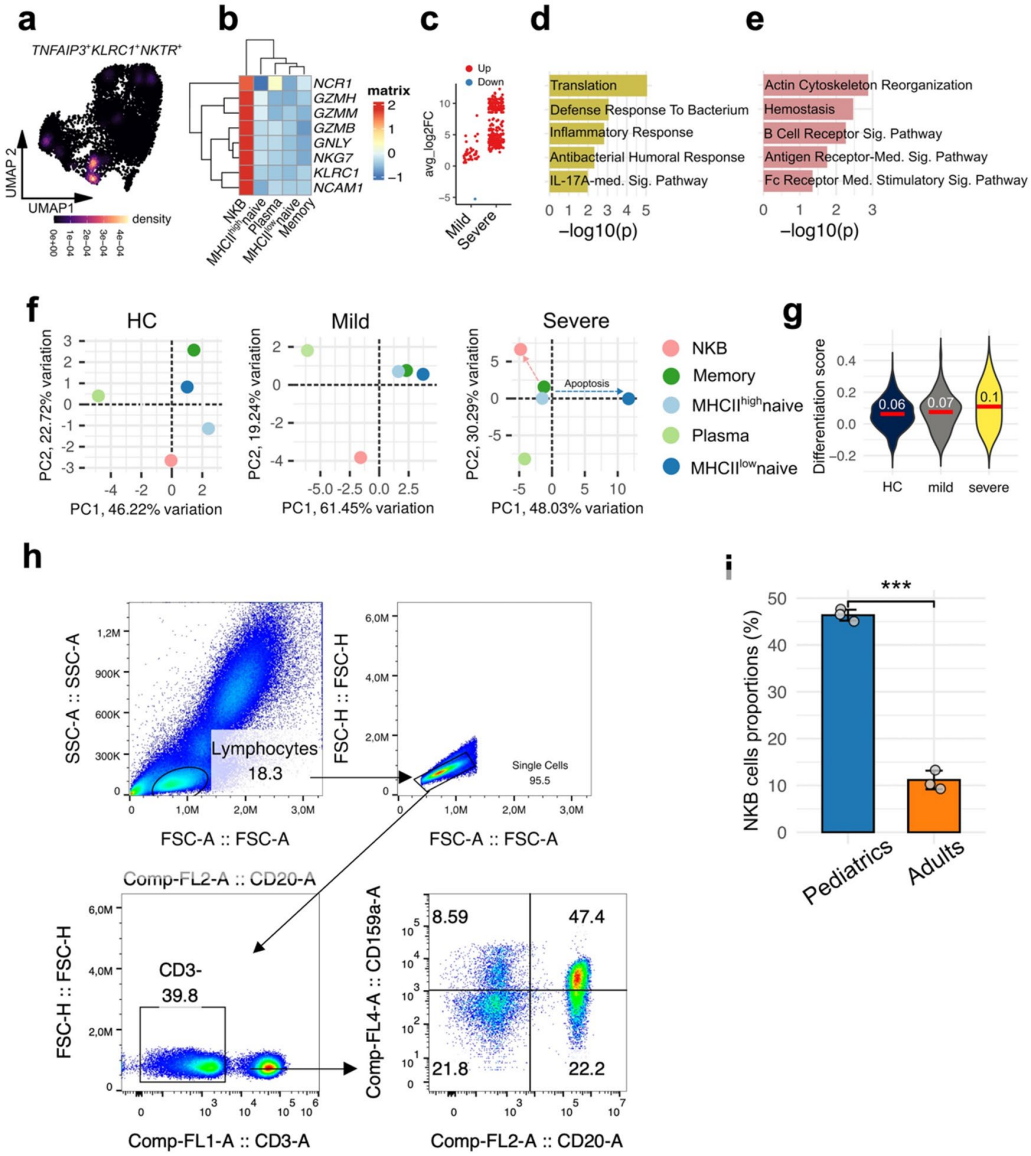
Cellular differentiation may shape the fate of NKB cells in pediatric severe sepsis. **a**. UMAP density plot illustrating the expression of canonical NKB cell marker genes. **b**. Heatmap showing the expression profiles of NK cell–associated genes across B cell subpopulations. **c**. Scatter plots displaying differentially expressed genes (DEGs) in severe and mild sepsis compared to healthy controls (highlighted in red and blue, respectively). **d–e**. Gene Ontology (GO) enrichment analysis of biological processes associated with DEGs in (**d**) mild sepsis and (**e**) severe sepsis. **f**. Principal component analysis (PCA) of the top 500 most variable genes across B cell subpopulations and study conditions. **g**. Box plot displaying B cell differentiation scores across the three clinical conditions. **h.** Flow cytometric (FACS) analysis of surface marker expression on whole blood from a healthy pediatric donor, showing the identification of natural killer-like B (NKB) cells defined as CD3⁻ CD20⁺ CD159a⁺. **i.** Bar plot displaying the proportion of NKB cells among total CD20⁺ B cells in peripheral blood from three adult and three pediatric donors. Individual data points are shown; error bars represent the standard deviation, *** *p* < 0.001.

The second most depleted B cell subpopulation in severe sepsis was the natural killer-like B (NKB) cells. These cells, express CD27 (**Supp.** Figure 1b), a marker usually attributed to memory cells^23^, but they are a distinct subset clustered far apart from Memory cells (Fig. 2a). Upon analyzing their gene expression profiles, we confirmed a distinct expression pattern compared to memory cells, associated with natural killer (NK) marker genes, including *KLRC1*, *NKTR*, *NCR1*, *GZMB*, and others (Fig. 4a–b and **Supp.** Figure 2a). Based on this unique transcriptional signature, we designated this subset as Natural Killer-like B (NKB) cells. In support, NKB cells have been reported before and it has been suggested that they derive from pro-B cells^24^, which also have been reported to express CD27^25^. By calculating the module score of NK cell–associated genes, we determined that NKB cells comprise approximately 45% of the entire CD27⁺ B-cell subpopulation (**Supp.** Figure 2a–b**)**.

NKB cells displayed a hybrid transcriptional signature combining NK effector molecules (*NKG7*, *GNLY*, *KLRD1*, and others) (**Supp.** Figure 2a), with B cell developmental factors (*TCF7*, *LEF1*, *SATB1*) and activation signaling components (*LCP1*, *LCP2*, *FYN*) (**Supp.** Figure 2c), indicating preserved cytotoxic capacity alongside adaptive immune function. Expression of inflammatory receptors (*IL6ST*) and homeostatic signals (*IL7R*) suggests these cells may contribute to both immune surveillance and sepsis-associated hyper-inflammation. Biological pathway analysis of NKB-associated genes revealed enrichment of natural killer cell-mediated cytotoxicity, acute inflammatory response, cellular response to reactive oxygen species (ROS), and interleukin-4 (IL-4) signaling pathways (**Supp.** Figure 2d), further supporting their role in both innate effector functions and inflammation during critical illness.

Similar to MHC II^high^ naïve B cells, NKB cells in severe sepsis exhibited a significantly higher number of upregulated genes compared to those in mild sepsis and healthy donors (Fig. 4c). Gene Ontology enrichment analysis revealed that these upregulated genes were associated with Fc receptor signaling pathways and actin cytoskeleton reorganization, suggesting a state of heightened activation and differentiation in NKB cells (Fig. 4e). In contrast, mild sepsis exhibited regular response to infection-associated pathways (Fig. 4d).

To further characterize these cells, we performed principal component analysis (PCA) using the top 500 highly variable genes across all B cell subtypes and clinical groups (Fig. 4e). The PCA revealed that NKB cells occupy a unique transcriptional space, shifting toward naïve and memory B cell compartments, a pattern consistent with active differentiation (Fig. 4f), which was further affirmed by the heightened B cell differentiation score in severe compared to mild sepsis as well as healthy donors (Fig. 4g).

Additionally, we inferred the presence of NKB cells in adult sepsis using GSE167363 dataset. In this cohort, CD27^+^KLRC1^+^ B-cells were considered NKB cells (**Supp.** Figure 3a–b). Similar to pediatric sepsis, adult non-survivors (matched with severe cases) showed a marked reduction in NKB cells compared to both survivors and healthy controls (**Supp.** Figure 3c), with Gene Ontology (GO) enrichment analysis indicating significant enrichment of positive regulation of lymphocyte differentiation in sepsis non-survivors (**Supp.** Figure 4a). When comparing NKB cells between pediatric and adult donors, pediatric NKB cells appeared to be in a developmental state, characterized by enrichment of pathways related to B cell differentiation, activation, proliferation, hematopoiesis, and chromatin remodeling. In contrast, adult NKB cells exhibited a more functionally mature immune profile, with upregulation of pathways associated with cytokine response, interferon-alpha signaling, and antiviral defense (**Supp.** Figure 4b). To validate the existence of NKB cells in peripheral blood, we performed flow cytometric analysis targeting the surface markers CD159a (*KLRC1*) and CD20 (*MS4A1*) in both pediatric and adult donors. Interestingly, NKB cells were substantially reduced in adults compared to children (Fig. 4h–i and **Supp.** Figure 5), indicating a possible age-dependent plasticity or regulation of this unique B cell subset.

**Fig. 5:**
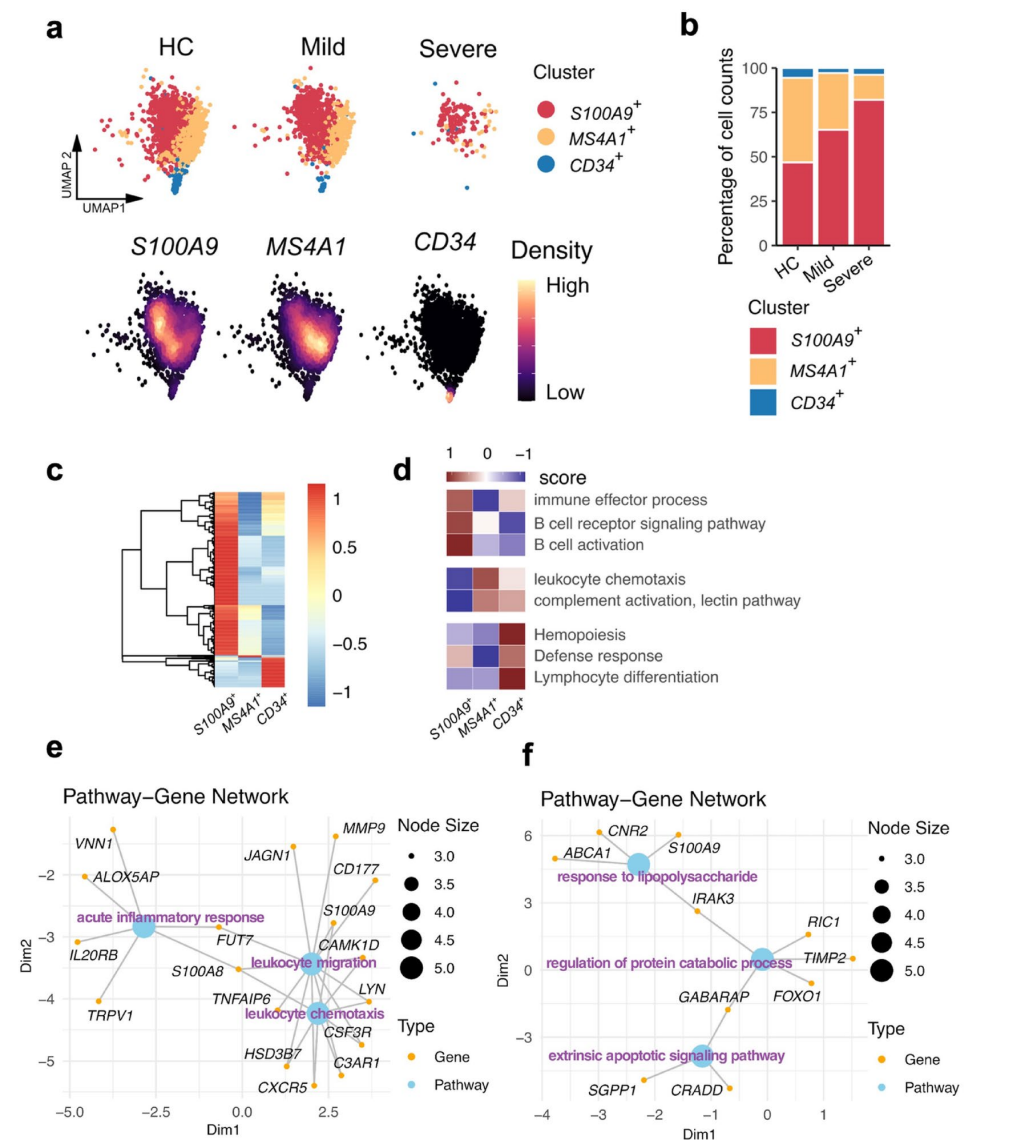
The reduction of MHC II^low^naïve B cells in severe pediatric sepsis may be driven by increased extrinsic apoptosis and cellular migration. **a**. UMAP visualization of MHC II^low^naïve B cells across the three study conditions, annotated by subpopulation. **b**. Bar plots showing differences in the relative abundance of MHC II^low^ naïve B cell subclusters across study groups. **c**. Heatmap of differentially expressed genes (DEGs) across MHC II^low^naïve B cell subclusters. **d**. Gene Ontology (GO) enrichment analysis of biological processes associated with DEGs identified in (**c**). **e–f**. Gene-pathway network analysis of DEGs in severe sepsis versus healthy controls for (**e**) CD20⁺ naïve B cells and (**f**) CD34⁺ naïve B cells.

### Extrinsic apoptosis and enhanced migratory activity could underlie the loss of MHC II^low^naïve B cells in severe pediatric sepsis

MHC II^low^naïve B cells were the third most depleted B cell subtype in individuals with severe sepsis. Through subclustering and annotation of the MHC II^low^naïve B cell compartment (Fig. 5a–b), we identified three major affected subpopulations: *CD34*⁺MHC II^low^naïve B cells, representing early B cell progenitors; *MS4A1*⁺MHC II^low^naïve B cells; and *S100A9*⁺MHC II^low^ naïve B cells. Differentially expressed gene (DEG) analysis using significant z-scores revealed 407 genes associated with *S100A9*⁺MHC II^low^ naïve B cells, 5 with *MS4A1*⁺MHC II^low^naïve B cells, and 75 with *CD34*⁺MHC II^low^ naïve B cells (Fig. 5c). These results suggest that the S100A9⁺ and CD20⁺ MHC II^low^naïve B-cell clusters represent mixed subpopulations. Notably, the *S100A9*⁺ subset exhibited the largest cell number (Fig. 5b) and the highest number of DEGs in severe sepsis (Fig. 5c).

GO enrichment analysis of DEGs from each MHC II^low^naïve subpopulation revealed distinct functional signatures: B cell activation in *S100A9*⁺, leukocyte chemotaxis in *MS4A1*⁺, and hematopoiesis in *CD34*⁺ MHC II^low^naïve B cells (Fig. 5d). These results suggest that each subset may respond differently to the immunopathological environment of severe sepsis.

To understand the mechanisms behind the marked depletion of *CD34*⁺ and *MS4A1*⁺ MHC II^low^naïve B cells, we curated DEGs between severe sepsis and healthy donors and constructed pathway gene interaction networks (Fig. 5e–f). The loss of *MS4A1*⁺MHC II^low^naïve B cells may be attributed to enhanced leukocyte migration (Fig. 5e), while extrinsic apoptotic signaling likely contributes to the reduction of *CD34*⁺ progenitor B cells (Fig. 5f). Collectively, these findings suggest a significant contraction of the MHC II^low^naïve B cell pool in severe sepsis, likely driven by extrinsic apoptosis and migratory responses, which may impair B cell replenishment and contribute to immune dysfunction in these patients.

### The crosstalk between B cells and neutrophils

Neutrophils were the only cells enriched in severe sepsis, and B-cells were significantly decreased with the higher discriminator power compared to the other cell populations. Knowing that B cells can affect neutrophil behavior^26,27^, we investigated if there is a crosstalk between neutrophils and B-cell subpopulations. In pediatric sepsis, our receptor–ligand interaction analysis revealed a selective CD45–CD22 signaling axis between memory B cells and neutrophils (**Supp. Figure 6a–b**). Additionally, memory B cells exhibited CD45–CD22 interactions with other B-cell subtypes, suggesting an inhibitory communication network within the B-cell compartment. CD22 acts as an inhibitory co-receptor that dampens BCR signaling, and when this negative signal is sufficiently strong, it can induce B-cell apoptosis^28^. This finding indicates that excessive CD45–CD22 engagement during sepsis may contribute to the suppression or loss of B-cell function.

In adult sepsis, we observed that all the B cell subtypes interacted with neutrophils, including NKB cells. We performed cell–cell interaction analysis, which revealed several signaling pathways that could potentially influence the progression of sepsis (**Supp. Figure 6c–d**). Communication probability scores highlighted the ANXA1–FPR1 axis and CD55-ADGRE5 axis key contributors to sepsis severity with their potential effects listed in Supp. Figure 6e.

## Discussion

Our current results showed that B cells have a pivotal role in disease progression and severity, as they exhibited the strongest discriminatory power in distinguishing severe from mild sepsis, and provided novel insights into the profound alterations in B cell subsets in severe pediatric sepsis, revealing the mechanisms of immune dysregulation and suggesting potential therapeutic targets. The pathogenesis of sepsis is driven by a complex interplay between pro-inflammatory and anti-inflammatory immune responses initiated by a wide range of pathogens, including bacteria, fungi, parasites, and viruses^9,16^. While neutrophils, the first responders to infection play a critical role in host defense by rapidly targeting and eliminating invading microbes^29,30^, which is in accordance with our previous work showing a unique shift in neutrophil subpopulations and functions as a predominant discriminator between acute and recovery phases in sepsis^16^, the loss of host adaptive immune function is considered a major cause of immunosuppression in sepsis. B cells, central components of adaptive immunity, help protect the host by producing pathogen-specific antibodies and modulating immune responses through cytokine secretion and interaction with other immune cells^31^. Severe sepsis is frequently associated with pronounced B cell lymphopenia^32^, and our data revealed that MHC II^high^naïve, NKB, and MHC II^low^naïve B cell subsets were particularly affected. The observed lymphopenia may arise from multiple mechanisms, including intrinsic or extrinsic apoptosis, cellular migration, and differentiation.

MHC II^high^naïve B cells are the major antigen-presenting cells (APCs) among B cells subsets^33–35^, which were notably depleted in severe sepsis. This is consistent with a previous report linking reduced MHC MHC II^high^naïve B cell counts to poor prognosis in sepsis non-survivors^36^. Transcriptomic profiling in our study indicated that MHC II^high^naïve B cells in severe sepsis undergo significant changes associated with intrinsic apoptosis. This was accompanied by upregulation of homeostatic pathways, suggesting a compensatory response to cellular depletion through cell death^37^. In line with this, one study reported that septic patients decreased Bcl-2 expression and increased levels of cleaved caspase-9 in circulating transitional B cells^38^, pointing toward intrinsic apoptosis as a key mechanism of loss.

NKB cells were also significantly depleted in severe sepsis. The identification of NKB cells, co-expressing canonical markers of both innate NK cells and adaptive B cells, highlights the potential significance of this rare B cell subset in pediatric sepsis. These cells displayed upregulation of cytotoxic-associated genes such as *KLRC1*, *NKTR*, *NCR1*, and *GZMB*, suggesting a functional shift toward innate cytotoxicity. NKB cells are a recently identified subset of innate lymphocytes that co-express markers of both NK cells (e.g., NKp46, NK1.1) and B cells (e.g., CD19, IgM)^39^. Found in lymphoid organs like the spleen and mesenteric lymph nodes, NKB cells are phenotypically and functionally distinct from conventional NK and B cells^40^. They are defined by their ability to rapidly secrete pro-inflammatory cytokines, particularly IL-18 and IL-12, in response to microbial infections^41,42^. NKB cells have been demonstrated to function as a separate subset of “innate-like B cells” (ILB)^24^. Substantial evidence supports the existence of ILB as multiple B cell subsets that are phenotypically and functionally distinct from conventional B cells, facilitating the innate immune response and serving as a bridge between the innate and adaptive components of the immune system^43–45^. ILB, particularly the B-1 and marginal zone (MZ) subsets, are the primary source of natural immunoglobulin (Ig)M production^29,46–48^. Among the similarities between NKB cells and conventional B cells is the expression of receptors and ligands with roles in antigen presentation and recognition, as well as class switching, affinity maturation, and B cell memory formation in secondary lymphoid follicles^49^. NKB cells primarily express IgD, and to a lesser extent IgM and IgG^49^. NKB cells also express NK cell activation receptors, Fas ligand, perforin, and granzymes and are capable of lysing cells^49,50^. Additionally, NKB cells produce the inflammatory cytokines, interferon gamma (IFN-γ) and tumor necrosis factor alpha (TNF-α). Finally, NKB cells have an increased proliferation capacity in comparison to NK and CD8^+^ T cells within Rhesus Macaques (RM) Simian Immunodeficiency Virus (SIV)-infected colon^51^. They have been also implicated in alcoholic liver disease, periodontal disease, hepatocellular carcinoma, and rheumatoid arthritis^39,50,52–54^. In these contexts, they promote inflammation through IL-18-dependent activation of neutrophils, NK cells, and CD8⁺ T cells, contributing to neutrophil-driven pathology and immune dysregulation. Despite their emerging importance, the precise developmental origins and lineage relationships of NKB cells remain under investigation, as does their full role in modulating adaptive immunity and disease pathogenesis. To our knowledge, this is the first study to report the presence of NKB cells in human peripheral blood, with their detection to be more prominent in pediatric populations due to their higher relative abundance compared to adults.

In addition, MHC II^low^naïve B cells, particularly *CD34*⁺ progenitors and *MS4A1*⁺ subsets, were markedly depleted in severe pediatric sepsis. As the earliest responders in the adaptive immune response, naïve B cells are activated upon encountering APCs^55^. Their reduction has previously been associated with prolonged sepsis and poor clinical outcomes^32^. In our data, transcriptomic alterations in both *CD34*⁺ progenitors and *MS4A1*⁺ subsets suggest that their depletion may be driven by extrinsic apoptosis and dysregulated chemotactic signaling. The loss of these subpopulations likely impairs the replenishment of the peripheral B cell pool, thereby exacerbating immune dysfunction.

Furthermore, neutrophils were the only cell population enriched in both mild and severe sepsis. Neutrophils play a major role in host defense and organ dysfunction in sepsis at early phase. It has been reported that B-cells can modulate neutrophil functions to promote disease tolerance, while neutrophils can influence B-cell survival and differentiation through cytokine release. Specifically, recent studies suggest that B-cells can influence the tissue-damaging properties of neutrophils during sepsis. In mouse models of severe sepsis, B-cells have been shown to be key regulators of tissue integrity. They can promote “disease tolerance” by suppressing the tissue-damaging effects of neutrophils, potentially by influencing neutrophil CXCR4 signaling^56^. This indicates that B-cells may play a protective role in limiting excessive neutrophil-mediated pathology in some septic contexts. Thus, less B-cells in severe sepsis can be associated with less protection from neutrophil damage. In addition, during inflammatory conditions, neutrophils can migrate to lymphoid organs where they engage in bidirectional interactions with B-cells (and T-cells). This can involve the release of cytokines such as B-cell activating factor (BAFF) and a proliferation-inducing ligand (APRIL) by neutrophils, which are crucial for plasma cell survival, development, proliferation, and differentiation into plasma cells^57–59^. In accordance, plasma cells were the only B-cell subpopulation expanded in severe sepsis in our cohort, where more neutrophils are expected. While the context of sepsis and acute inflammation is distinct from chronic autoimmune diseases like rheumatoid arthritis (RA), some insights into B-cell/neutrophil crosstalk from RA may be relevant. In RA, a “vicious circle” develops where pathogenic autoantibodies (e.g., anti-citrullinated protein antibodies, ACPAs) produced by autoreactive B-cells form immune complexes that potently activate neutrophils^60^. These activated neutrophils then release pro-inflammatory mediators and neutrophil extracellular traps (NETs), which can further generate citrullinated epitopes, contributing to renewed ACPA production by B-cells and perpetuating inflammation^61–63^. These autoreactive B cells are generally memory B cells or plasma cells that derive from germinal center reactions in lymphoid tissues (e.g., lymph nodes, ectopic lymphoid structures in synovium). A subset of class-switched memory B cells and CD138⁺ plasma cells are known to secrete ACPAs as well. This also aligns with our results, since plasma cells and neutrophils where the only cells enriched in severe sepsis. In addition, neutrophils can also suppress B-cell activity under certain conditions. For example, they may inhibit IgA production or germinal center formation via mechanisms like TGF-β1 secretion or reactive oxygen species, which could also explain our results.

Moreover, when we performed B cell-neutrophil interaction analysis, we confirmed an interaction between memory B cells and neutrophils. CD45-CD22 pathway was the main pathway for this interaction. CD22 on memory cells serve as an inhibitory signal for BCR via CD45^64,65^. It is possible that abundant neutrophils in sepsis could impair memory B cell function via this pathway in pediatric sepsis. Interestingly, we observed much more diverse interactions between neutrophils and B cells subpopulations in adult sepsis. NKB cells were one of them. CD55 on B cells interacted with ADGRE5 on neutrophils. This interaction is known to regulate the complement pathway. However, the detailed biological role of this pathway in neutrophils-B cell interaction needs further investigation^66^. In addition, NKB cells interacted with neutrophils via ANXA1–FPR1. In general, ANXA1 expression is considered low in B cells, but it is interesting to determine if NKB cells have unique ANXA1 expression profile. Whether or not pediatric NKB cells have similar interaction with neutrophils need future study.

## Conclusions

This study highlights the role of B cells in the progression of severe sepsis, reveals the presence of NKB cells in human circulation, and uncovers multiple mechanisms contributing to peripheral B cell loss, including intrinsic or extrinsic apoptosis, migration, and differentiation, antigen presentation and cross-talking with neutrophils. The observed alterations across B cell subsets underscore the urgency for therapeutic strategies that preserve B cell function and survival during severe immune challenges.

Targeted interventions aimed at inhibiting apoptotic pathways or enhancing B cell resilience may help counteract sepsis-induced immunosuppression. It may also help managing neutrophil-mediated inflammation and organ damage. Even more specifically, targeted approaches aiming to either deplete plasma cells, that seem to be proinflammatory and may activate neutrophils, or approaches to enhance the identified B cell subpopulations (NKB, MHC II^high^naïve B cells, MHC II^low^naïve B cells) that could control neutrophil-mediated damage could be proven beneficial for septic patients. Furthermore, the identification of NKB cells opens new avenues for research into unconventional B cell subsets and their roles in host defense.

In conclusion, our findings provide a comprehensive view of B cell dysregulation in severe pediatric sepsis, illustrating a dynamic interplay between cell depletion, subset emergence, and immune competency. These insights offer a valuable framework for developing targeted therapies to restore immune balance and improve patient outcomes.

## Supplementary Information

Below is the link to the electronic supplementary material.

Supplementary Material 1

## Data Availability

The single-cell RNA-Seq datasets generated in this study have been deposited in Zenodo under the identifier 10.1016/j.clim.2024.110175.

## Abbreviations

AP: Acute phase
APCs: Antigen-presenting cells
AUC: Area under the curve
BCR: B cell receptor
DEGs: Differentially expressed genes
ILB: Innate-like B cells
ICUs: Intensive care units
NKB: Natural killer-like B cells
scRNA-seq: Single-cell RNA sequencing
PICUs: Pediatric intensive care units
PSOFA: Pediatric sequential organ failure assessment
PCA: Principal component analysis
ROC: Receiver operating characteristic
RP: Recovery phase
