# Supplementary material for: Single-cell analysis of B cell dysregulation in pediatric sepsis stratified by disease severity
Source: Sci Rep. 2025 Dec 31;16:3987. doi: 10.1038/s41598-025-34126-9 (PMC12855920; doi:10.1038/s41598-025-34126-9)
Supplement: Supplementary file 1 — Supplementary Material 1 [file 41598_2025_34126_MOESM1_ESM.pdf]

Supplementary Table 1: Absolute cell counts per donor.

Subject	Total cells	B cells
Severe 1	3,560	67
Severe 2	1,779	12
Severe 3	8,805	721
Mild 1	4,429	1,929
Mild 2	4,952	31
Mild 3	4,155	634
Healthy 1	2,789	357
Healthy 2	14,347	2,189
Healthy 3	2,633	408
Healthy 4	4,788	1,602

Supp. Fig. 1. Dot plot showing the expression of representative marker genes for (**a**) major peripheral blood immune cell types and (**b**) B cell subpopulations.

a

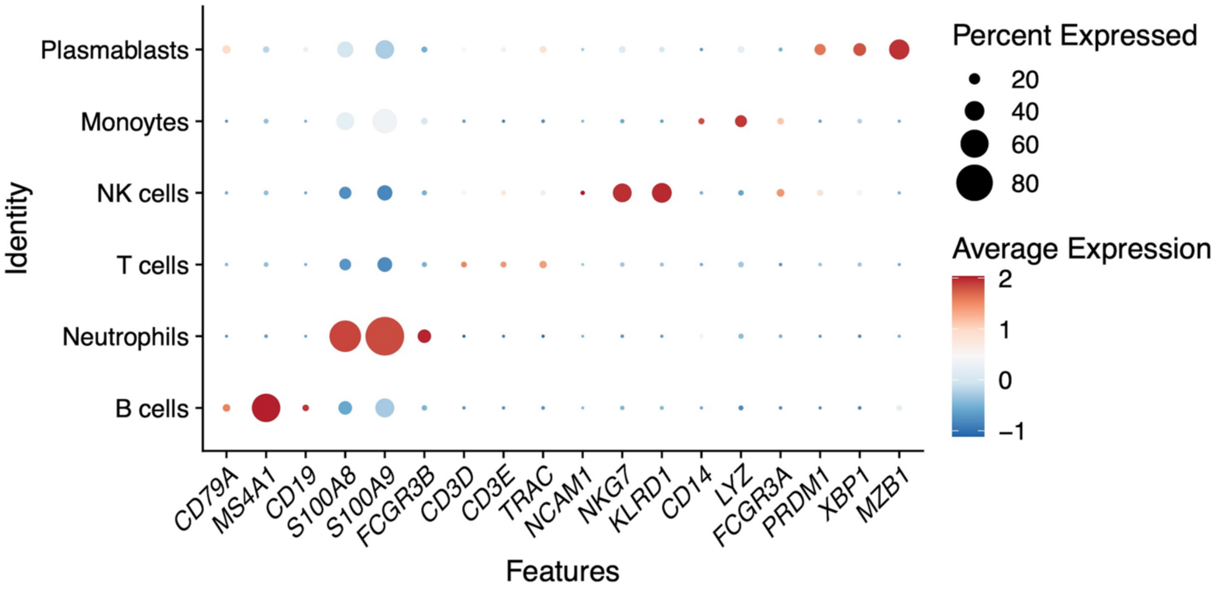

b

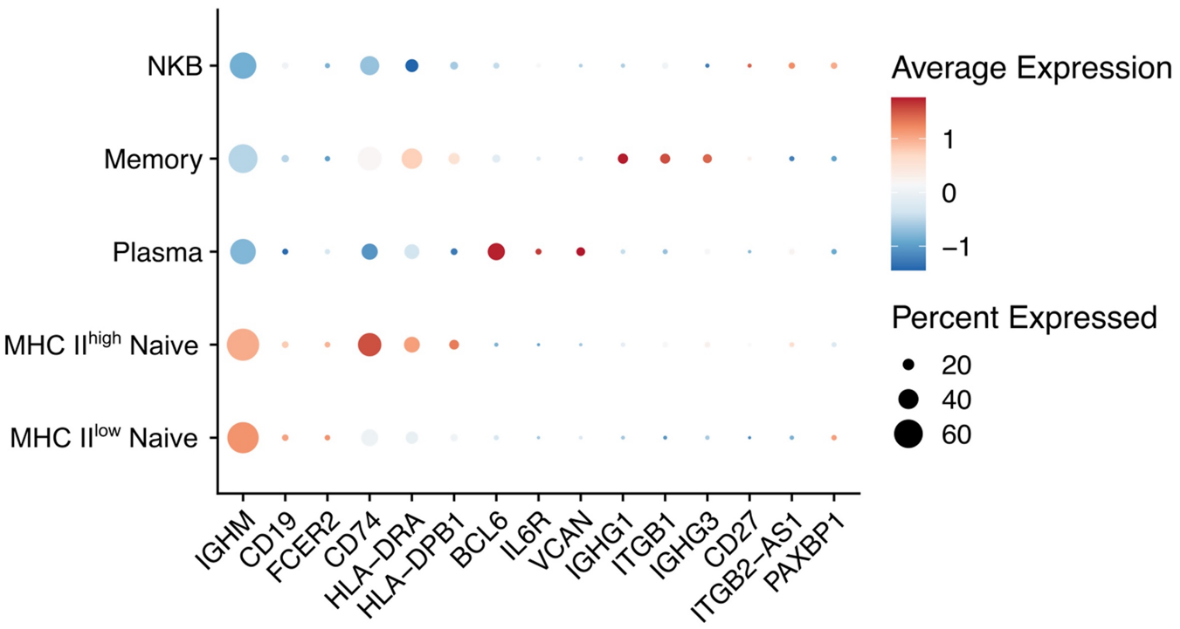

Supp. Fig. 2. (**a**) Dot plot showing the expression of NK-associated genes for NKB cells with (**b**) their proportion of NKB cells expressing these gene within the total CD27+ memory subpopulation. (**c**) Gene signatures selectively expressed by NKB cells compared to the rest of the other B cell subpopulations. (**d**) Biological pathway analysis of genes enriched within NKB cells.

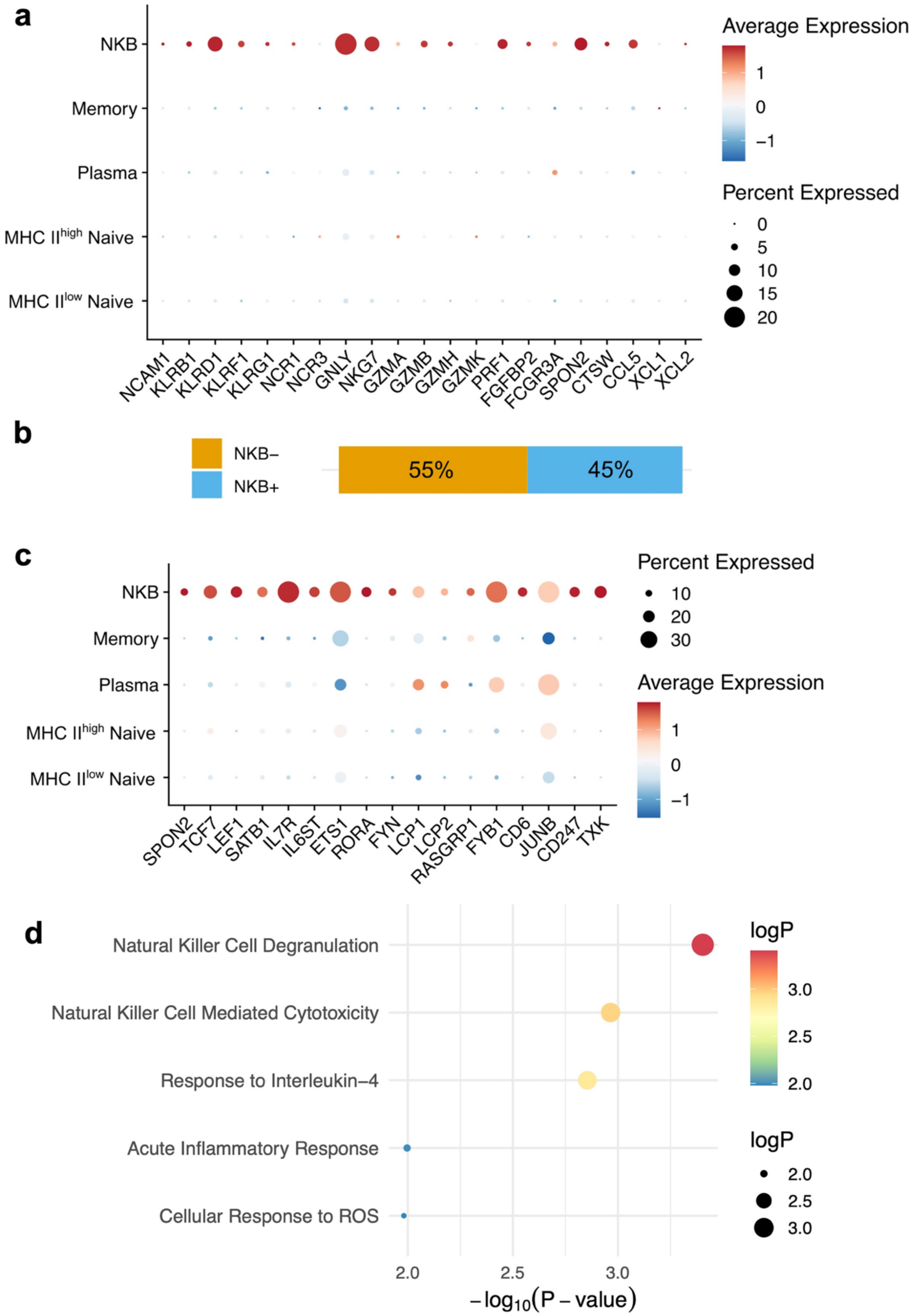

Supp. Fig. 3. NKB cells characterization in adult sepsis.

a, UMAP visualization of B cells from adult sepsis patients (2 healthy controls, 3 survivors, and 2 non-survivors; GEO: GSE167363).

b, UMAP density plot illustrating the expression of KLRC1 (canonical NKB cell marker gene).

c, Boxplots showing the relative abundance of each NKB cell proportion across the three study groups, HC: Healthy Controls; S: Survivors; NS: Non-Survivors.

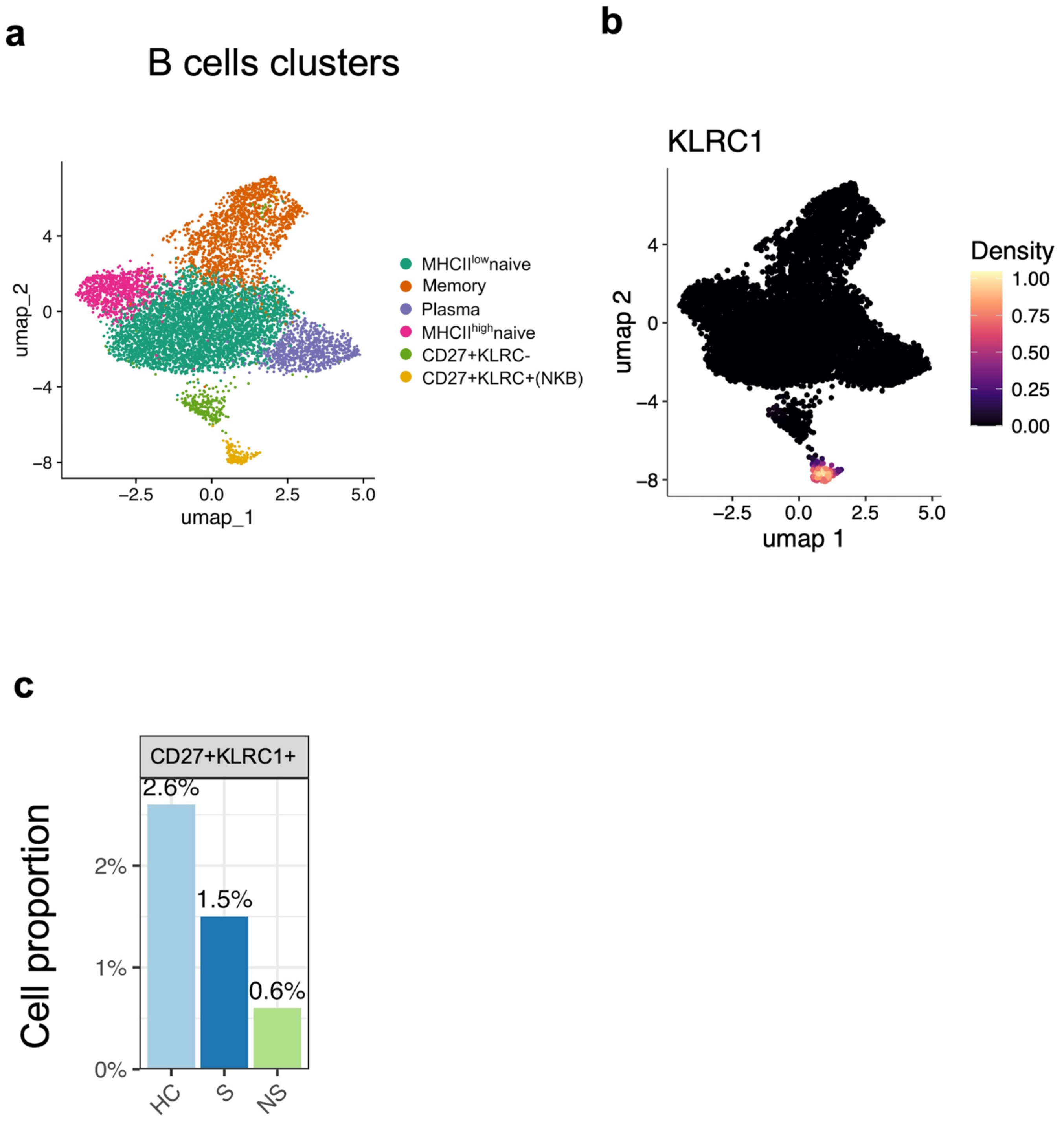

Supp. Fig. 4. Gene Ontology (GO) enrichment analysis of biological processes associated with DEGs of NKB cells in (**a**) sepsis survivors and non-survivors, and (**b**) adult versus pediatric sepsis.

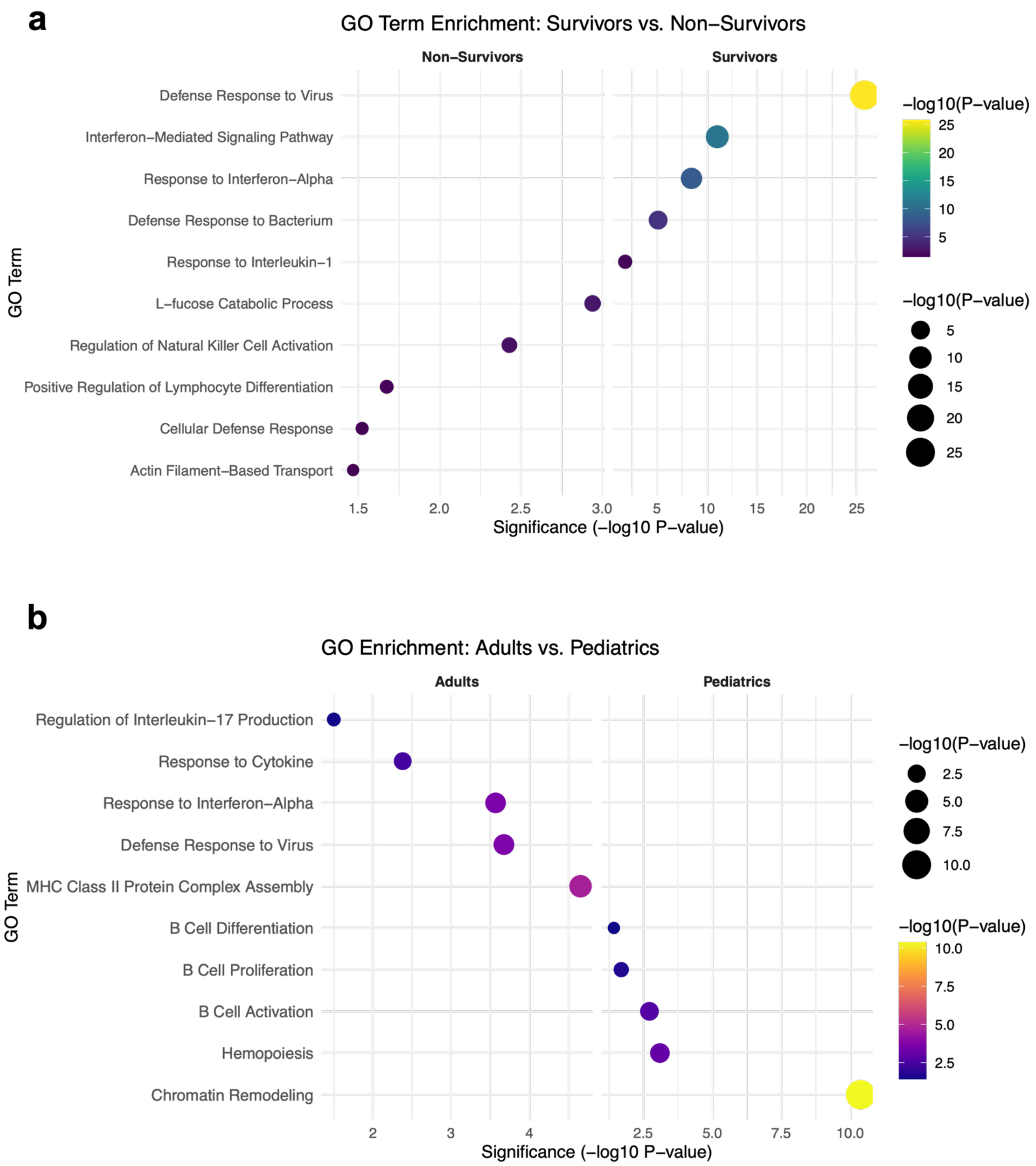

Supp. Fig. 5. Flow cytometric (FACS) analysis of surface marker expression on whole blood from a healthy adult donor, showing the identification of natural killer-like B (NKB) cells defined as CD3⁻ CD20⁺ CD159a⁺.

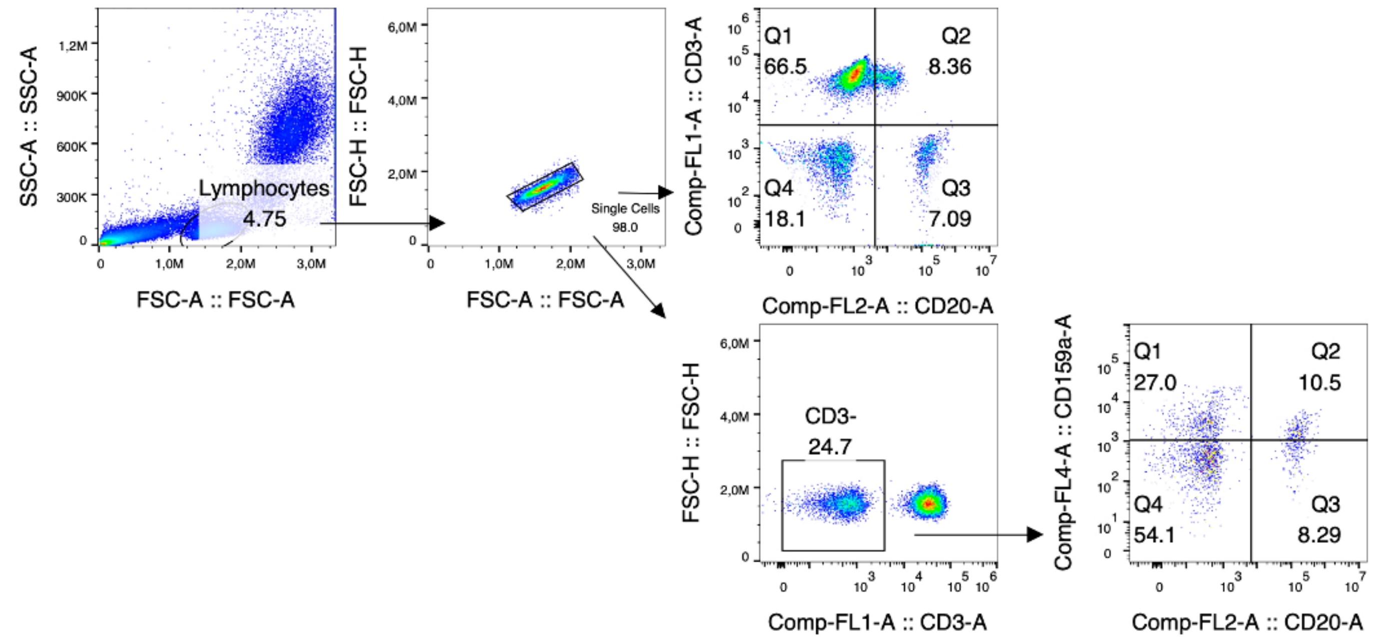

Supp. Fig. 6. (**a**), Cell-cell interaction analysis depicting various receptor-ligand interactions between NKB cells and the rest of B cell subsets and neutrophils in pediatric sepsis cohort with (**b**) dot plot showing their corresponding significance values. (**c–d**) Dot plots depicting the cell-cell interaction pathways between B cell subpopulations and neutrophils in adult sepsis. (**e**) Literature research on the role of different NKB cell – Neutrophil interaction pathways and their contribution score to sepsis severity.

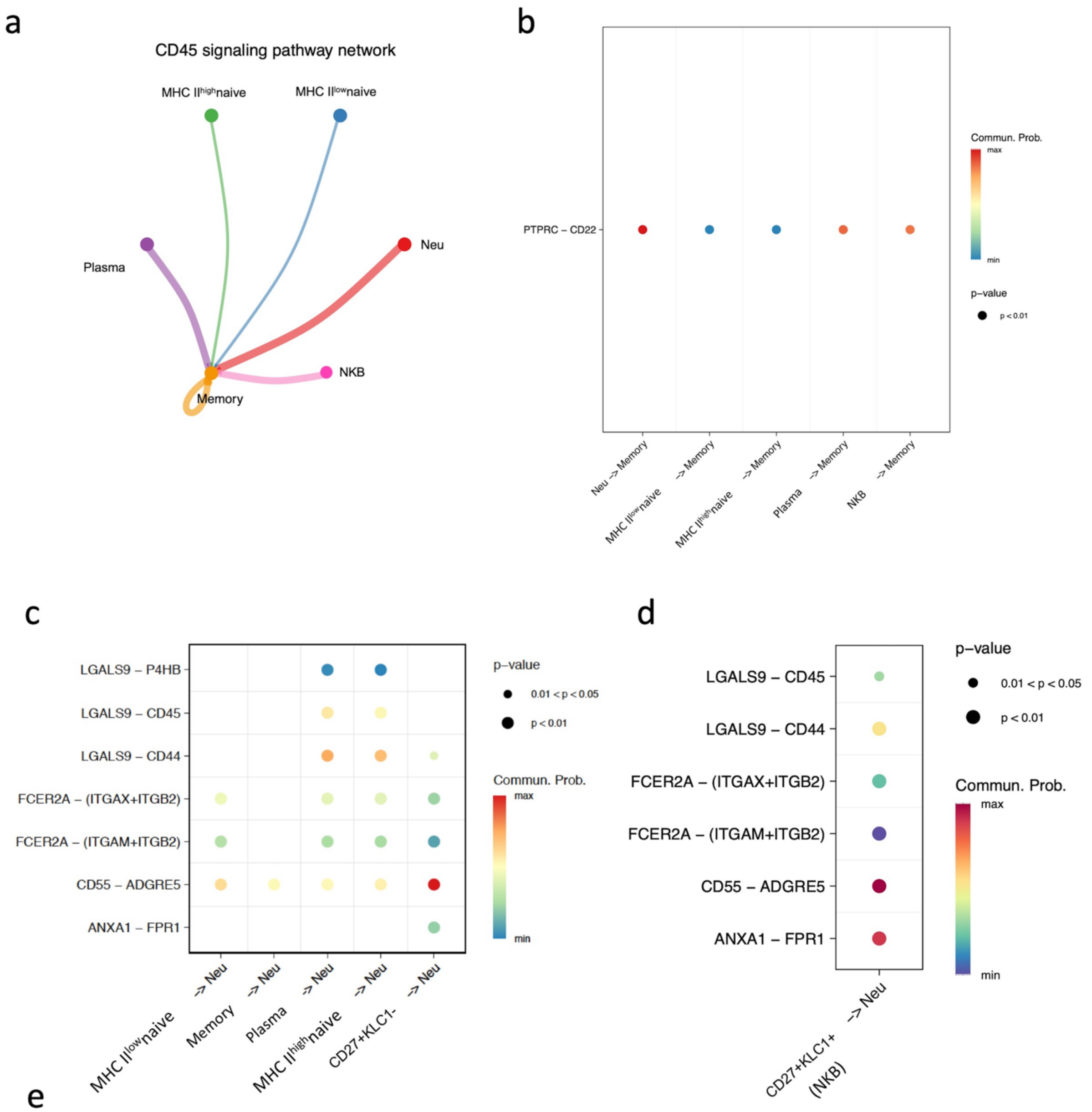

e

Pathway	Mechanism
ANXA1 – FPR1	Failure of resolution, neutrophil overactivation
LGALS9 – CD44	Immunosuppression, neutrophil retention
FCER2A – (ITGAM+ITGB2)	Neutrophil recruitment, hyperactivation
LGALS9 – CD45	Neutrophil functional inhibition
CD55 – ADGRE5	Neutrophil trafficking, survival
FCER2A – (ITGAX+ITGB2)	Less common, immune complex signaling
